# Supplementary material for: Integrated platform for structural and functional analysis of terpene synthases of Cannabis sativa
Source: PeerJ. 2025 Jul 10;13:e19723. doi: 10.7717/peerj.19723 (PMC12256043; doi:10.7717/peerj.19723)
Supplement: Supplemental Information 1 — All conditions used in thermal melts and crystallization screen and all raw data for protein expression, HPLC and thermofluor assay. [file peerj-13-19723-s001.docx]

**Table S1.** Buffers and additives used in the thermofluor protein stability screen

| No. | Buffer | Additive | No. | Buffer | Additive |
| --- | --- | --- | --- | --- | --- |
| 1 | 0.2 M MES (pH 5.5) | - | 41 | 0.2 M HEPES (pH 7.5) | - |
| 2 | 0.2 M MES (pH 5.5) | 0.05 M NaCl | 42 | 0.2 M HEPES (pH 7.5) | 0.05 M NaCl |
| 3 | 0.2 M MES (pH 5.5) | 0.1 M NaCl | 43 | 0.2 M HEPES (pH 7.5) | 0.1 M NaCl |
| 4 | 0.2 M MES (pH 5.5) | 0.2 M NaCl | 44 | 0.2 M HEPES (pH 7.5) | 0.2 M NaCl |
| 5 | 0.2 M MES (pH 5.5) | 0.05 M KCl | 45 | 0.2 M HEPES (pH 7.5) | 0.05 M KCl |
| 6 | 0.2 M MES (pH 5.5) | 0.1 M KCl | 46 | 0.2 M HEPES (pH 7.5) | 0.1 M KCl |
| 7 | 0.2 M MES (pH 5.5) | 0.2 M KCl | 47 | 0.2 M HEPES (pH 7.5) | 0.2 M KCl |
| 8 | 0.2 M MES (pH 5.5) | 0.05 M MgCl^2+^ | 48 | 0.2 M HEPES (pH 7.5) | 0.05 M MgCl^2+^ |
| 9 | 0.2 M MES (pH 5.5) | 0.1 M MgCl^2+^ | 49 | 0.2 M HEPES (pH 7.5) | 0.1 M MgCl^2+^ |
| 10 | 0.2 M MES (pH 5.5) | 0.2 M MgCl^2+^ | 50 | 0.2 M HEPES (pH 7.5) | 0.2 M MgCl^2+^ |
| 11 | 0.2 M Bis-Tris (pH 6.0) | - | 51 | 0.2 M Tris (pH 8.0) | - |
| 12 | 0.2 M Bis-Tris (pH 6.0) | 0.05 M NaCl | 52 | 0.2 M Tris (pH 8.0) | 0.05 M NaCl |
| 13 | 0.2 M Bis-Tris (pH 6.0) | 0.1 M NaCl | 53 | 0.2 M Tris (pH 8.0) | 0.1 M NaCl |
| 14 | 0.2 M Bis-Tris (pH 6.0) | 0.2 M NaCl | 54 | 0.2 M Tris (pH 8.0) | 0.2 M NaCl |
| 15 | 0.2 M Bis-Tris (pH 6.0) | 0.05 M KCl | 55 | 0.2 M Tris (pH 8.0) | 0.05 M KCl |
| 16 | 0.2 M Bis-Tris (pH 6.0) | 0.1 M KCl | 56 | 0.2 M Tris (pH 8.0) | 0.1 M KCl |
| 17 | 0.2 M Bis-Tris (pH 6.0) | 0.2 M KCl | 57 | 0.2 M Tris (pH 8.0) | 0.2 M KCl |
| 18 | 0.2 M Bis-Tris (pH 6.0) | 0.05 M MgCl^2+^ | 58 | 0.2 M Tris (pH 8.0) | 0.05 M MgCl^2+^ |
| 19 | 0.2 M Bis-Tris (pH 6.0) | 0.1 M MgCl^2+^ | 59 | 0.2 M Tris (pH 8.0) | 0.1 M MgCl^2+^ |
| 20 | 0.2 M Bis-Tris (pH 6.0) | 0.2 M MgCl^2+^ | 60 | 0.2 M Tris (pH 8.0) | 0.2 M MgCl^2+^ |
| 21 | 0.2 M Bis-Tris (pH 6.5) | - | 61 | 0.2 M Tris (pH 8.5) | - |
| 22 | 0.2 M Bis-Tris (pH 6.5) | 0.05 M NaCl | 62 | 0.2 M Tris (pH 8.5) | 0.05 M NaCl |
| 23 | 0.2 M Bis-Tris (pH 6.5) | 0.1 M NaCl | 63 | 0.2 M Tris (pH 8.5) | 0.1 M NaCl |
| 24 | 0.2 M Bis-Tris (pH 6.5) | 0.2 M NaCl | 64 | 0.2 M Tris (pH 8.5) | 0.2 M NaCl |
| 25 | 0.2 M Bis-Tris (pH 6.5) | 0.05 M KCl | 65 | 0.2 M Tris (pH 8.5) | 0.05 M KCl |
| 26 | 0.2 M Bis-Tris (pH 6.5) | 0.1 M KCl | 66 | 0.2 M Tris (pH 8.5) | 0.1 M KCl |
| 27 | 0.2 M Bis-Tris (pH 6.5) | 0.2 M KCl | 67 | 0.2 M Tris (pH 8.5) | 0.2 M KCl |
| 28 | 0.2 M Bis-Tris (pH 6.5) | 0.05 M MgCl^2+^ | 68 | 0.2 M Tris (pH 8.5) | 0.05 M MgCl^2+^ |
| 29 | 0.2 M Bis-Tris (pH 6.5) | 0.1 M MgCl^2+^ | 69 | 0.2 M Tris (pH 8.5) | 0.1 M MgCl^2+^ |
| 30 | 0.2 M Bis-Tris (pH 6.5) | 0.2 M MgCl^2+^ | 70 | 0.2 M Tris (pH 8.5) | 0.2 M MgCl^2+^ |
| 31 | 0.2 M HEPES (pH 7.0) | - | 71 | 0.2 M CAPS (pH 9.0) | - |
| 32 | 0.2 M HEPES (pH 7.0) | 0.05 M NaCl | 72 | 0.2 M CAPS (pH 9.0) | 0.05 M NaCl |
| 33 | 0.2 M HEPES (pH 7.0) | 0.1 M NaCl | 73 | 0.2 M CAPS (pH 9.0) | 0.1 M NaCl |
| 34 | 0.2 M HEPES (pH 7.0) | 0.2 M NaCl | 74 | 0.2 M CAPS (pH 9.0) | 0.2 M NaCl |
| 35 | 0.2 M HEPES (pH 7.0) | 0.05 M KCl | 75 | 0.2 M CAPS (pH 9.0) | 0.05 M KCl |
| 36 | 0.2 M HEPES (pH 7.0) | 0.1 M KCl | 76 | 0.2 M CAPS (pH 9.0) | 0.1 M KCl |
| 37 | 0.2 M HEPES (pH 7.0) | 0.2 M KCl | 77 | 0.2 M CAPS (pH 9.0) | 0.2 M KCl |
| 38 | 0.2 M HEPES (pH 7.0) | 0.05 M MgCl^2+^ | 78 | 0.2 M CAPS (pH 9.0) | 0.05 M MgCl^2+^ |
| 39 | 0.2 M HEPES (pH 7.0) | 0.1 M MgCl^2+^ | 79 | 0.2 M CAPS (pH 9.0) | 0.1 M MgCl^2+^ |
| 40 | 0.2 M HEPES (pH 7.0) | 0.2 M MgCl^2+^ | 80 | 0.2 M CAPS (pH 9.0) | 0.2 M MgCl^2+^ |

**Table S2**. Directed crystallisation screen for terpene synthase proteins.

| No. | Precipitant | Buffer | Salt |
| --- | --- | --- | --- |
| 1 | 0.1 M Bis-Tris (pH 6.0) | 5% PEG-3350 | 0.2 M NaCl, 0.2 M MgCl^2+^ |
| 2 | 0.1 M Bis-Tris (pH 6.5) | 5% PEG-3350 | 0.2 M NaCl, 0.2 M MgCl^2+^ |
| 3 | 0.1 M Bis-Tris (pH 7.0) | 5% PEG-3350 | 0.2 M NaCl, 0.2 M MgCl^2+^ |
| 4 | 0.1 M Tris (pH 7.5) | 5% PEG-3350 | 0.2 M NaCl, 0.2 M MgCl^2+^ |
| 5 | 0.1 M Tris (pH 8.0) | 5% PEG-3350 | 0.2 M NaCl, 0.2 M MgCl^2+^ |
| 6 | 0.1 M Tris (pH 8.5) | 5% PEG-3350 | 0.2 M NaCl, 0.2 M MgCl^2+^ |
| 7 | 0.1 M Bis-Tris (pH 6.0) | 15% PEG-3350 | 0.2 M NaCl, 0.2 M MgCl^2+^ |
| 9 | 0.1 M Bis-Tris (pH 6.5) | 15% PEG-3350 | 0.2 M NaCl, 0.2 M MgCl^2+^ |
| 10 | 0.1 M Bis-Tris (pH 7.0) | 15% PEG-3350 | 0.2 M NaCl, 0.2 M MgCl^2+^ |
| 11 | 0.1 M Tris (pH 7.5) | 15% PEG-3350 | 0.2 M NaCl, 0.2 M MgCl^2+^ |
| 12 | 0.1 M Tris (pH 8.0) | 15% PEG-3350 | 0.2 M NaCl, 0.2 M MgCl^2+^ |
| 13 | 0.1 M Tris (pH 8.5) | 15% PEG-3350 | 0.2 M NaCl, 0.2 M MgCl^2+^ |
| 14 | 0.1 M Bis-Tris (pH 6.0) | 25% PEG-3350 | 0.2 M NaCl, 0.2 M MgCl^2+^ |
| 15 | 0.1 M Bis-Tris (pH 6.5) | 25% PEG-3350 | 0.2 M NaCl, 0.2 M MgCl^2+^ |
| 16 | 0.1 M Bis-Tris (pH 7.0) | 25% PEG-3350 | 0.2 M NaCl, 0.2 M MgCl^2+^ |
| 17 | 0.1 M Tris (pH 7.5) | 25% PEG-3350 | 0.2 M NaCl, 0.2 M MgCl^2+^ |
| 18 | 0.1 M Tris (pH 8.0) | 25% PEG-3350 | 0.2 M NaCl, 0.2 M MgCl^2+^ |
| 19 | 0.1 M Tris (pH 8.5) | 25% PEG-3350 | 0.2 M NaCl, 0.2 M MgCl^2+^ |
| 20 | 0.1 M Bis-Tris (pH 6.0) | 35% PEG-3350 | 0.2 M NaCl, 0.2 M MgCl^2+^ |
| 21 | 0.1 M Bis-Tris (pH 6.5) | 35% PEG-3350 | 0.2 M NaCl, 0.2 M MgCl^2+^ |
| 22 | 0.1 M Bis-Tris (pH 7.0) | 35% PEG-3350 | 0.2 M NaCl, 0.2 M MgCl^2+^ |
| 23 | 0.1 M Tris (pH 7.5) | 35% PEG-3350 | 0.2 M NaCl, 0.2 M MgCl^2+^ |
| 24 | 0.1 M Tris (pH 8.0) | 35% PEG-3350 | 0.2 M NaCl, 0.2 M MgCl^2+^ |
| 25 | 0.1 M Bis-Tris (pH 6.0) | 5% PEG-8000 | 0.2 M NaCl, 0.2 M MgCl^2+^ |
| 26 | 0.1 M Bis-Tris (pH 6.5) | 5% PEG-8000 | 0.2 M NaCl, 0.2 M MgCl^2+^ |
| 27 | 0.1 M Bis-Tris (pH 7.0) | 5% PEG-8000 | 0.2 M NaCl, 0.2 M MgCl^2+^ |
| 28 | 0.1 M Tris (pH 7.5) | 5% PEG-8000 | 0.2 M NaCl, 0.2 M MgCl^2+^ |
| 29 | 0.1 M Tris (pH 8.0) | 5% PEG-8000 | 0.2 M NaCl, 0.2 M MgCl^2+^ |
| 30 | 0.1 M Tris (pH 8.5) | 5% PEG-8000 | 0.2 M NaCl, 0.2 M MgCl^2+^ |
| 31 | 0.1 M Bis-Tris (pH 6.0) | 15% PEG-8000 | 0.2 M NaCl, 0.2 M MgCl^2+^ |
| 32 | 0.1 M Bis-Tris (pH 6.5) | 15% PEG-8000 | 0.2 M NaCl, 0.2 M MgCl^2+^ |
| 33 | 0.1 M Bis-Tris (pH 7.0) | 15% PEG-8000 | 0.2 M NaCl, 0.2 M MgCl^2+^ |
| 34 | 0.1 M Tris (pH 7.5) | 15% PEG-8000 | 0.2 M NaCl, 0.2 M MgCl^2+^ |
| 35 | 0.1 M Tris (pH 8.0) | 15% PEG-8000 | 0.2 M NaCl, 0.2 M MgCl^2+^ |
| 36 | 0.1 M Tris (pH 8.5) | 15% PEG-8000 | 0.2 M NaCl, 0.2 M MgCl^2+^ |
| 37 | 0.1 M Bis-Tris (pH 6.0) | 25% PEG-8000 | 0.2 M NaCl, 0.2 M MgCl^2+^ |
| 38 | 0.1 M Bis-Tris (pH 6.5) | 25% PEG-8000 | 0.2 M NaCl, 0.2 M MgCl^2+^ |
| 39 | 0.1 M Bis-Tris (pH 7.0) | 25% PEG-8000 | 0.2 M NaCl, 0.2 M MgCl^2+^ |
| 40 | 0.1 M Tris (pH 7.5) | 25% PEG-8000 | 0.2 M NaCl, 0.2 M MgCl^2+^ |
| 41 | 0.1 M Tris (pH 8.0) | 25% PEG-8000 | 0.2 M NaCl, 0.2 M MgCl^2+^ |
| 42 | 0.1 M Tris (pH 8.5) | 25% PEG-8000 | 0.2 M NaCl, 0.2 M MgCl^2+^ |
| 43 | 0.1 M Bis-Tris (pH 6.0) | 35% PEG-8000 | 0.2 M NaCl, 0.2 M MgCl^2+^ |
| 44 | 0.1 M Bis-Tris (pH 6.5) | 35% PEG-8000 | 0.2 M NaCl, 0.2 M MgCl^2+^ |
| 45 | 0.1 M Bis-Tris (pH 7.0) | 35% PEG-8000 | 0.2 M NaCl, 0.2 M MgCl^2+^ |
| 46 | 0.1 M Tris (pH 7.5) | 35% PEG-8000 | 0.2 M NaCl, 0.2 M MgCl^2+^ |
| 47 | 0.1 M Tris (pH 8.0) | 35% PEG-8000 | 0.2 M NaCl, 0.2 M MgCl^2+^ |
| 48 | 0.1 M Tris (pH 8.5) | 35% PEG-8000 | 0.2 M NaCl, 0.2 M MgCl^2^ |


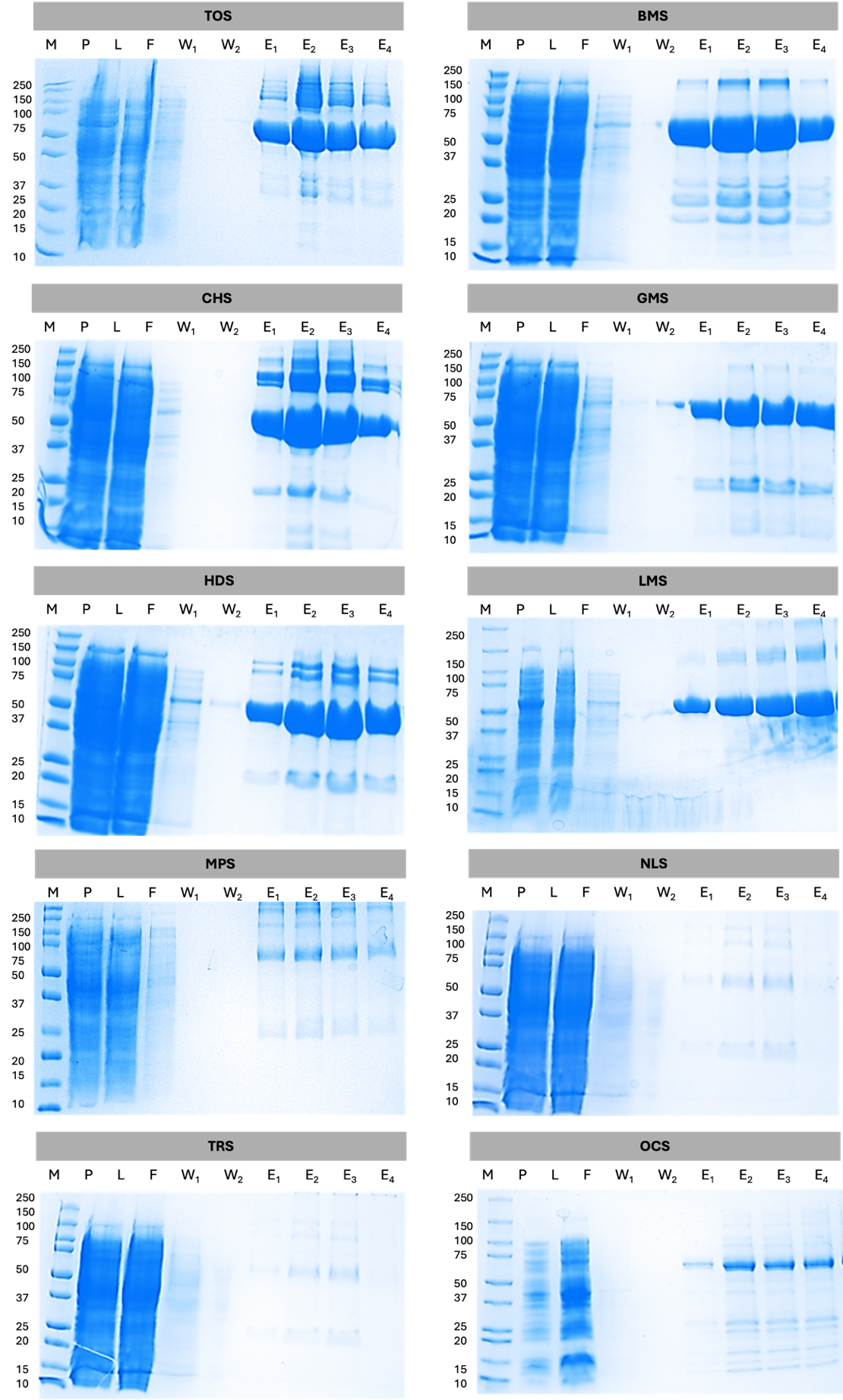


**Figure S1.** Purification of CsTPS proteins via Ni-NTA chromatography, visualised by 12% SDS-PAGE. The cell pellet (P) was collected after protein extraction for analysis, while the clarified cell lysate (L) was loaded onto a 2 mL Ni-NTA resin column. Proteins with intact hexahistidine tags were retained on the resin, and non-binding proteins were collected in the flow-through (F). The resin was washed with 10 mM imidazole (W1-2), and the bound protein was eluted in four 1 mL fractions (E1-4) using 200 mM imidazole.


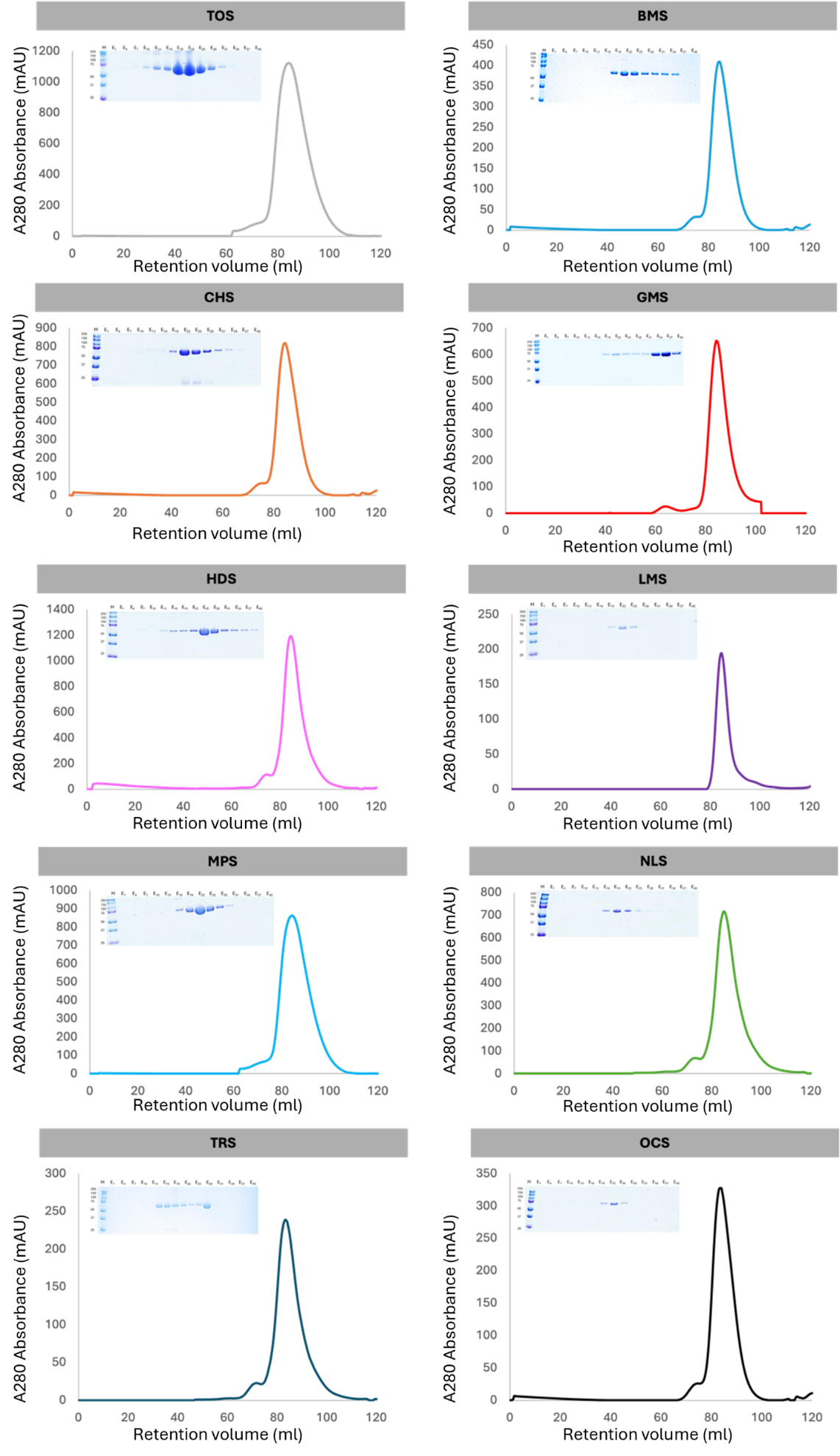


**Figure S2.** Size-exclusion chromatogram and correlating 12% SDS-PAGE of all CsTPS proteins. Purification was conducted via Superdex S200 16/60 chromatography column (GE Healthcare). Molecular weight markers are indicated in kDa.

***
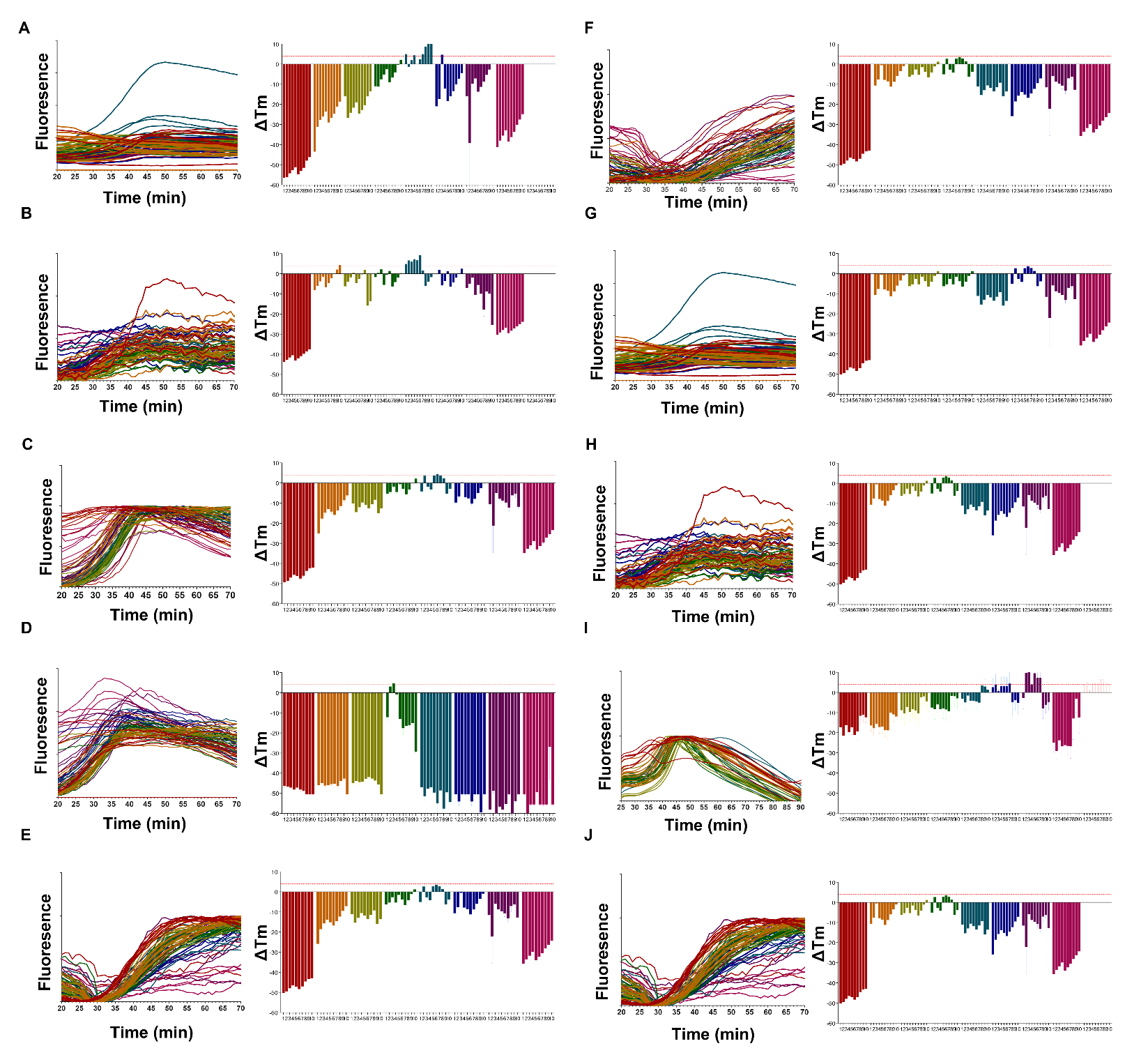
***

**Figure S3.** A. Thermal shift assay result for the initial screen of 10 CsTPS proteins. B. Changes in the unfolding transition temperature (∆T _m_) were calculated for measurement on 10 proteins in 56 buffer conditions. The bars represent the median ∆T _m_ values. A negative ∆T _m_ value signifies that the buffer destabilises the proteins, and a positive ∆T _m_ value indicates that the buffer has a stabilising effect.

| **CsTPS** | **Retention Time (min)** | | **Identified Terpene Compound** | **NIST Compound** | | **Head to Tail Comparison** | | |
| --- | --- | --- | --- | --- | --- | --- | --- | --- |
| CsTPS3FN | | 24.05 | β-myrcene | | 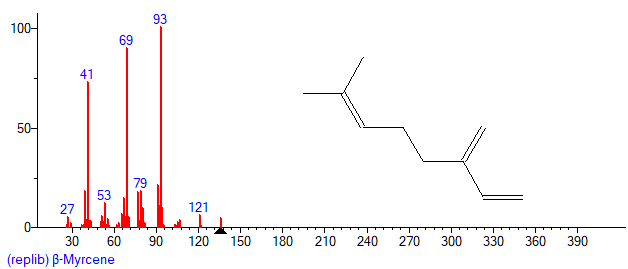 | | 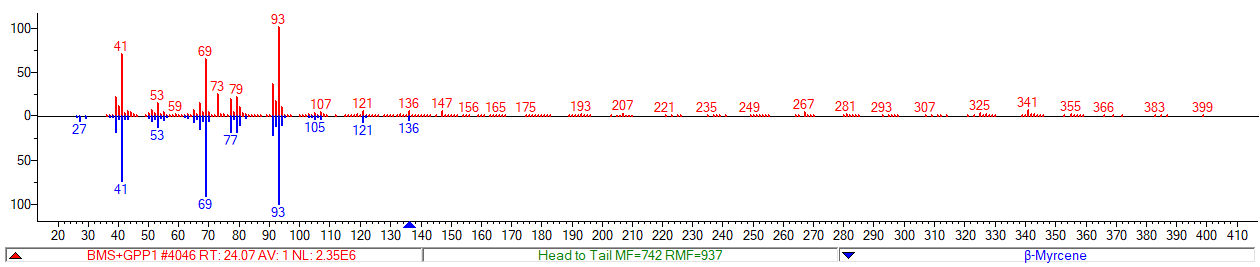 |  |
| CsTPS9FN | | 38.75 | β-caryophyllene | | 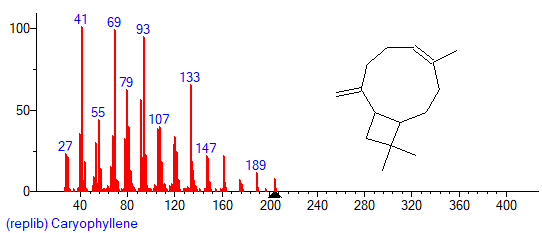 | | 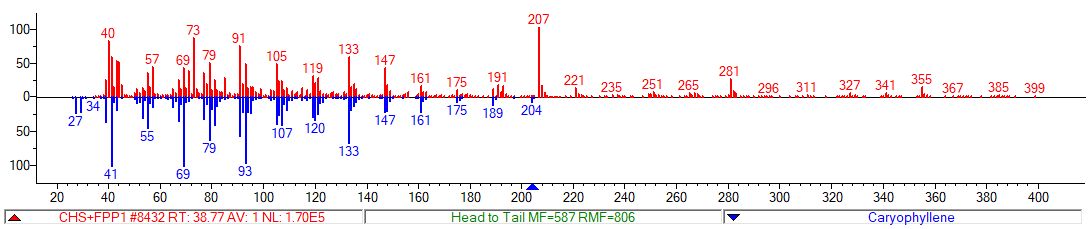 |  |
|  |  | 39.7 | humulene | | 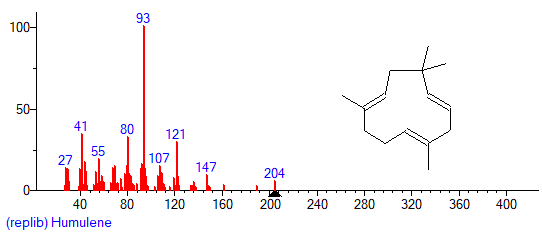 | | 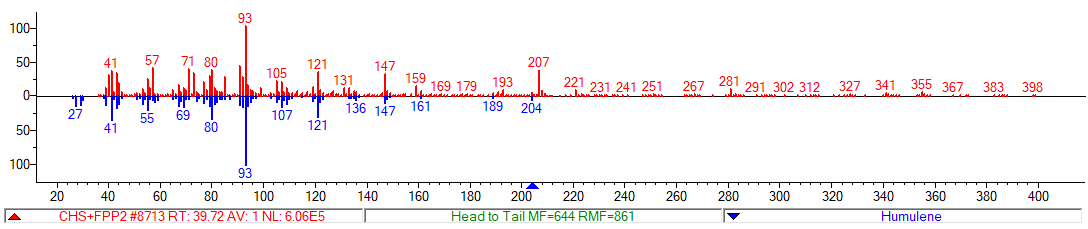 |  |
|  |  | 39.87 | epi-β-caryophyllene* | | 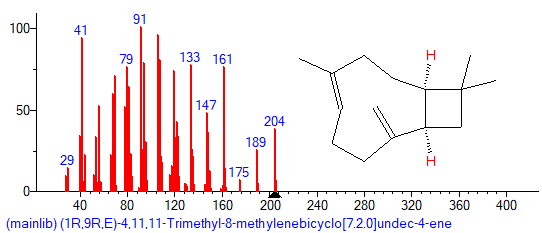 | | 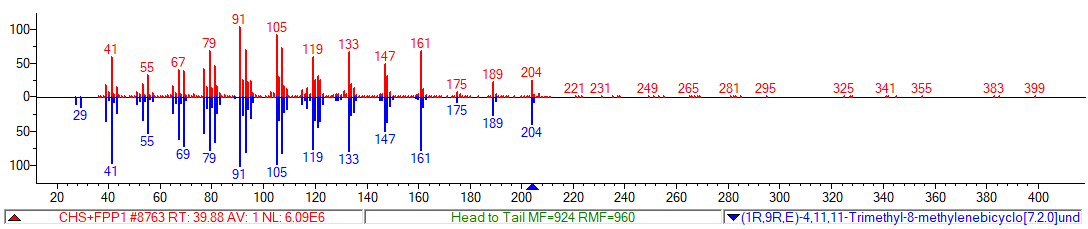 |  |
|  |  | 42.71 | germacrene D* | | 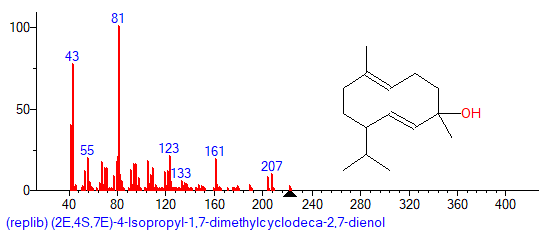 | | 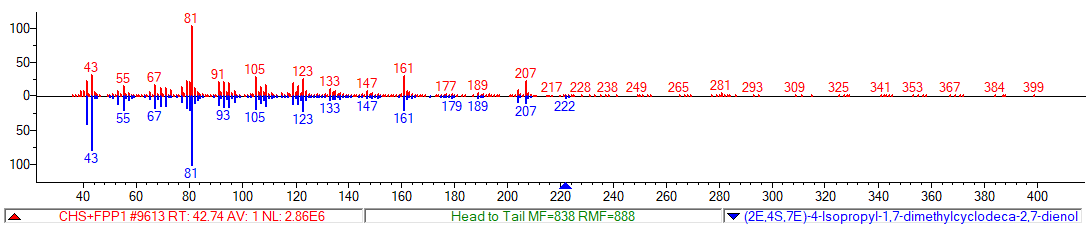 |  |
|  |  | 43.64 | globulol* | | 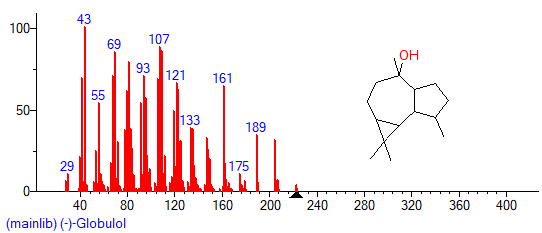 | | 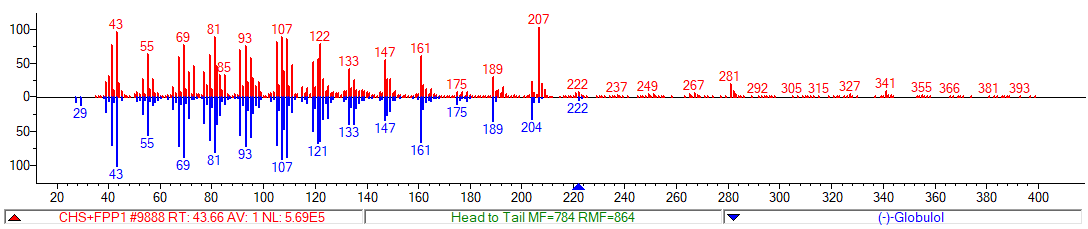 |  |
| CsTPS16CC | | 35.89 | δ-Eiemene* | | 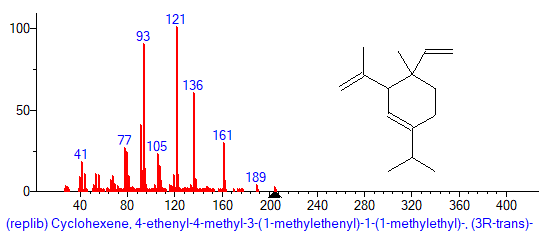 | | 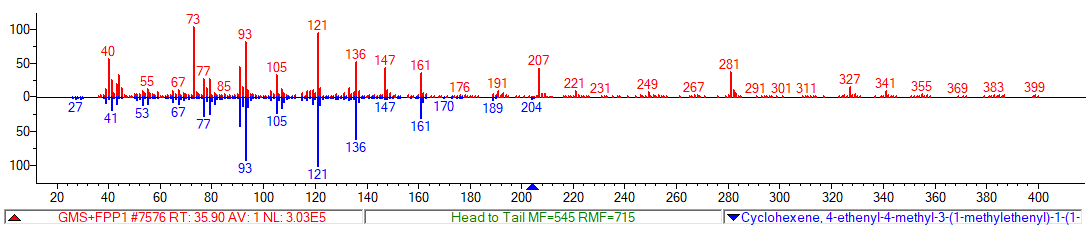 |  |
|  |  | 37.48 | β-Elemene* | | 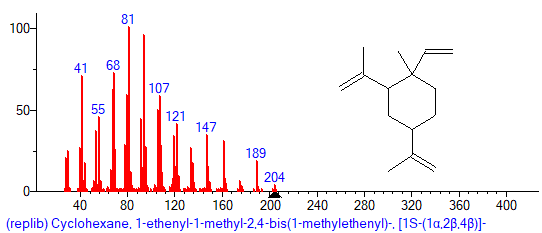 | | 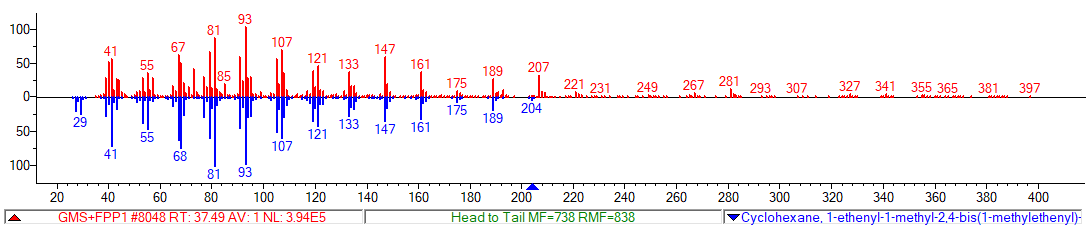 |  |
|  |  | 38.55 | y-elemene* | | 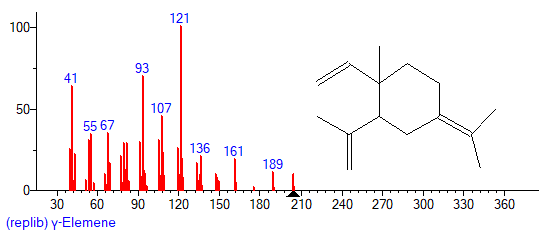 | | 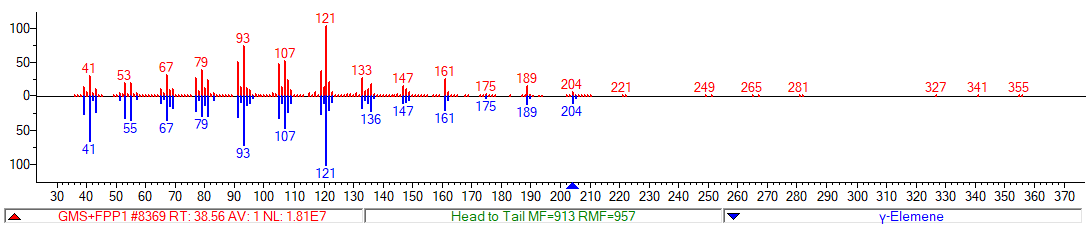 |  |
|  |  | 40.3 | alloaromadendrene* | | 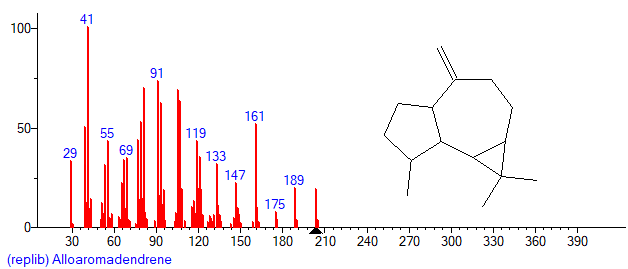 | | 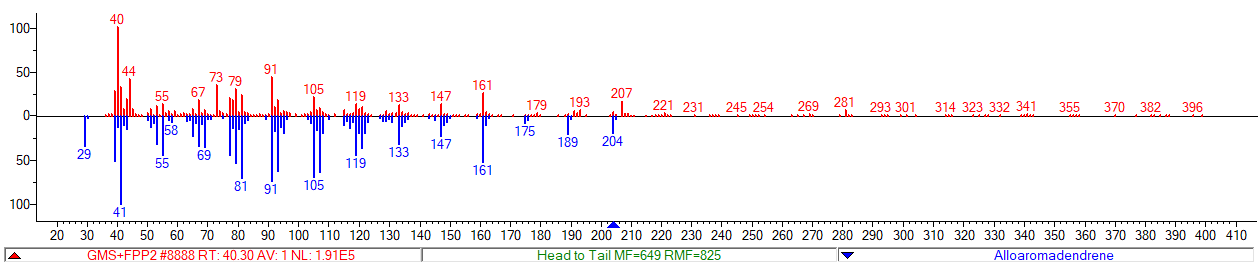 |  |
| CsTPS20CT | | 24.06 | β-myrcene | | 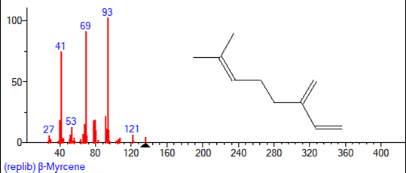 | | 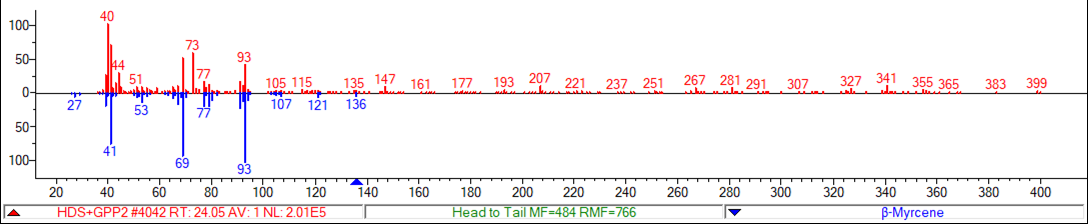 |  |
|  |  | 25.91 | limonene | | 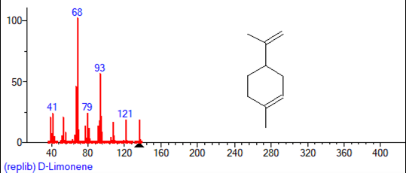 | | 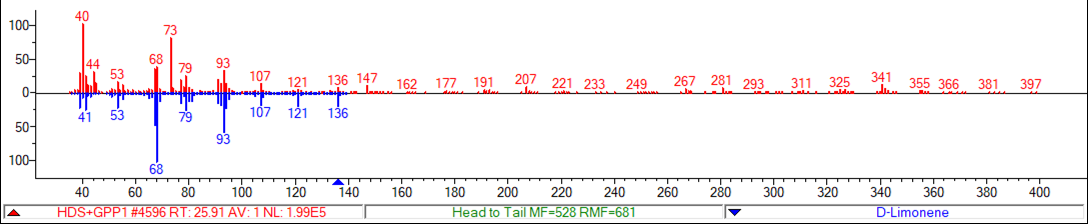 |  |
|  |  | 26.11 | (Z)-β-ocimene | |  | |  |  |
|  |  | 27.9 | terpinolene | | 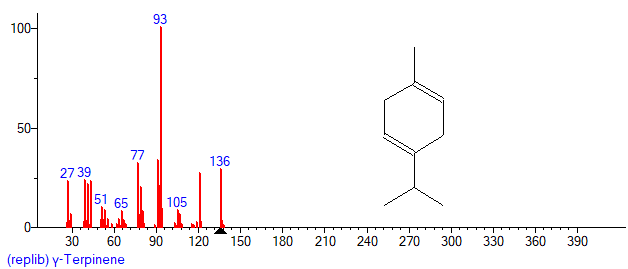 | | 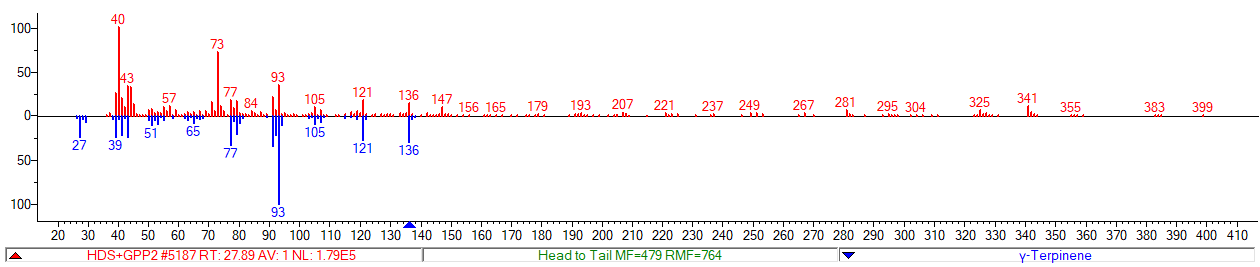 |  |
|  |  | 31.6 | α-terpineol | | 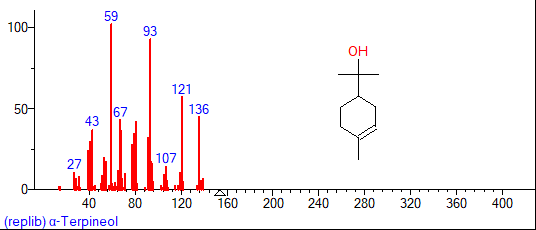 | | 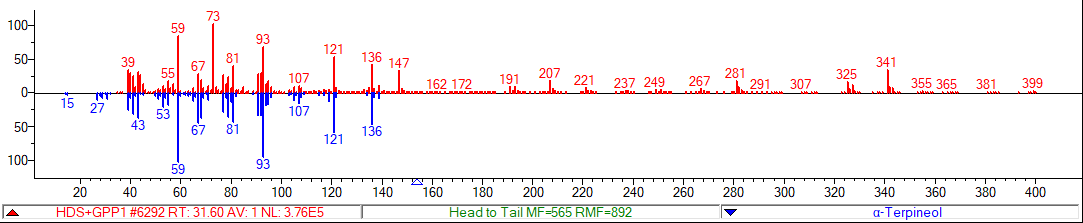 |  |
|  |  | 32.8 | geraniol | | 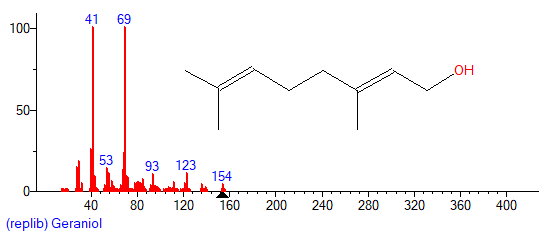 | | 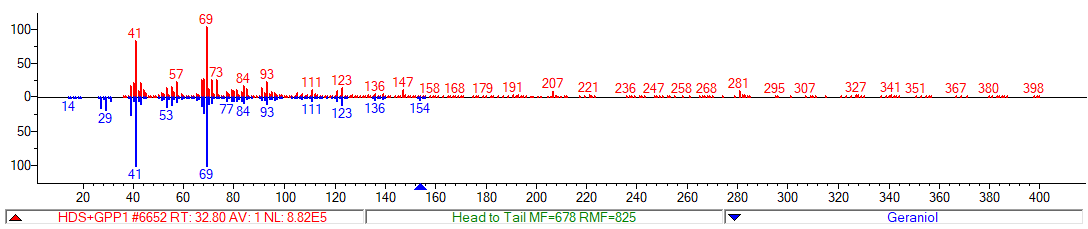 |  |
|  |  | 41.7 | elemol* | | 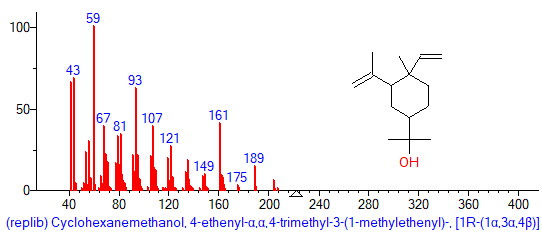 | | 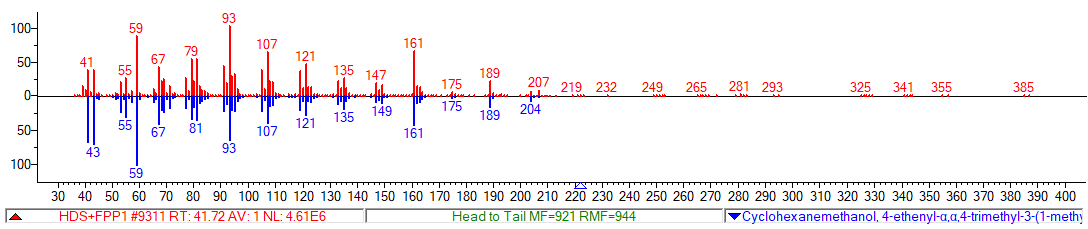 |  |
|  |  | 43.03 | guaiol | | 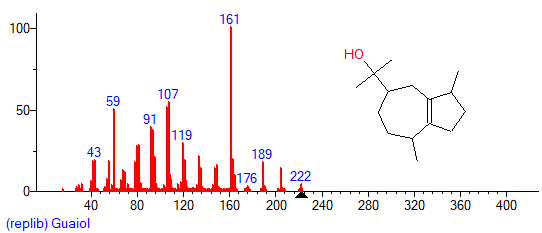 | | 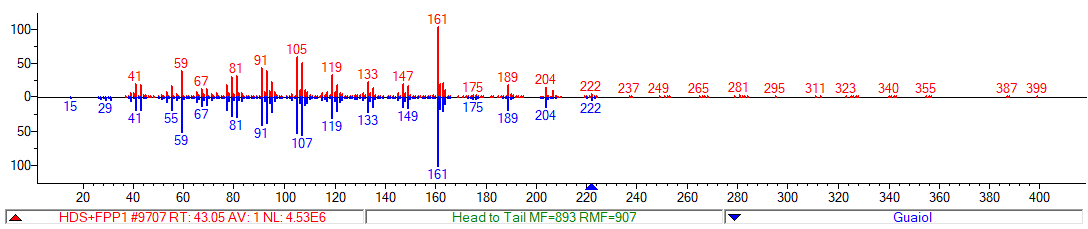 |  |
|  |  | 44.03 | γ-eudesmol* | | 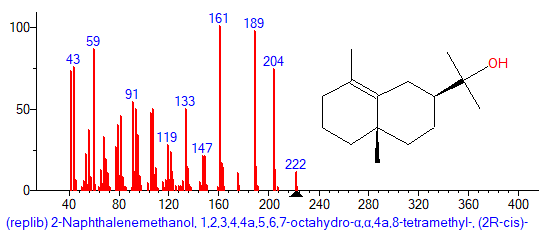 | | 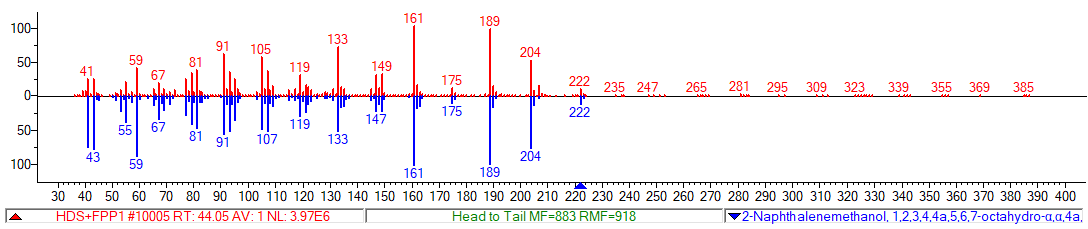 |  |
|  |  | 44.79 | α-eudesmol* | | 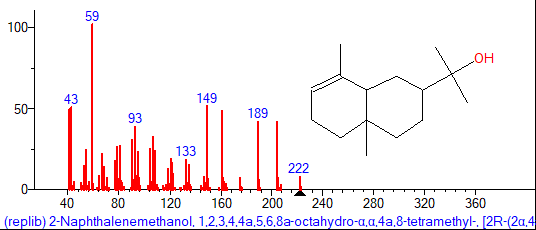 | | 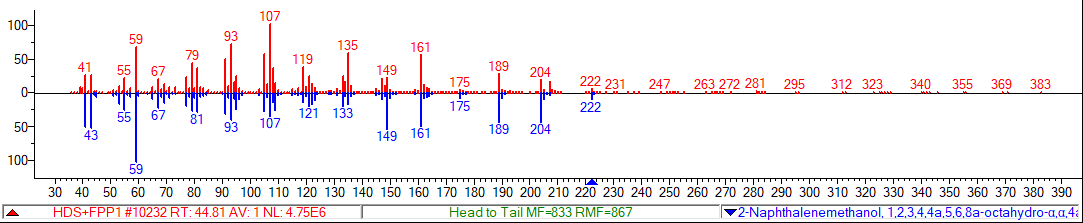 |  |
| CsTPS1SK | | 22.41 | α-pinene | | 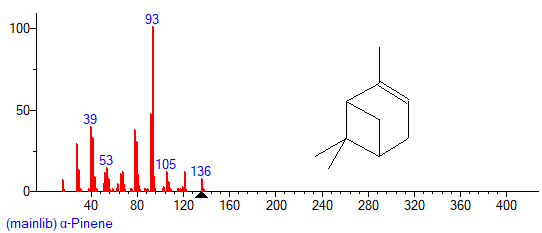 | | 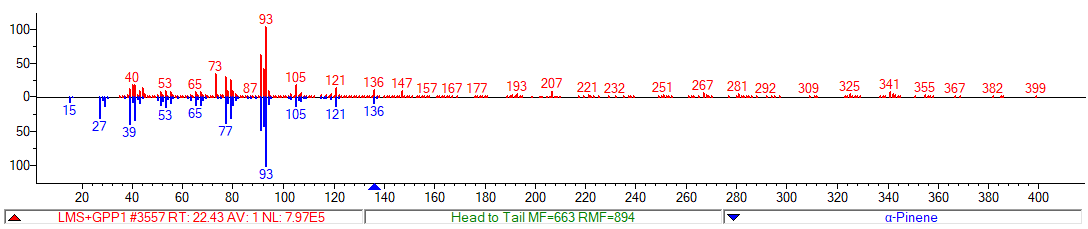 |  |
|  |  | 23.18 | camphene | | 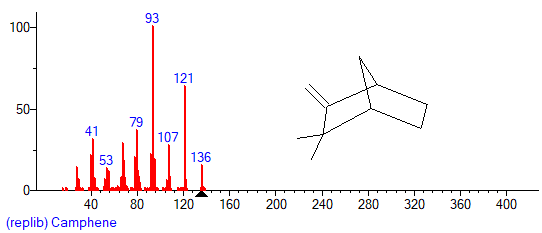 | | 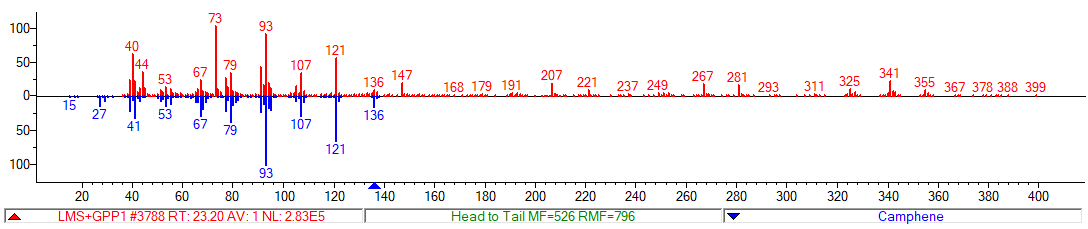 |  |
|  |  | 24.05 | β-myrcene | | 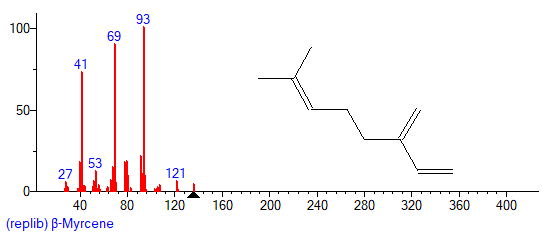 | | 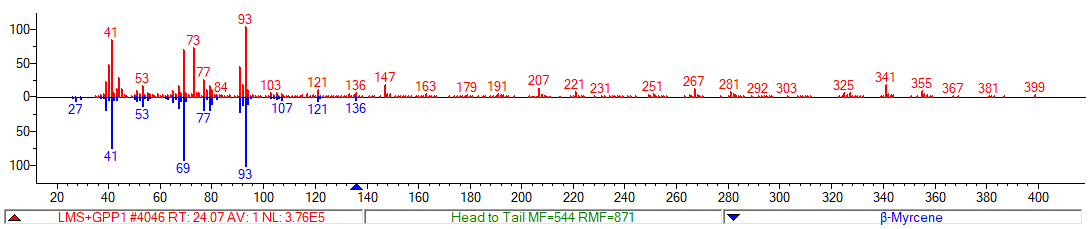 |  |
|  |  | 24.24 | β-pinene | | 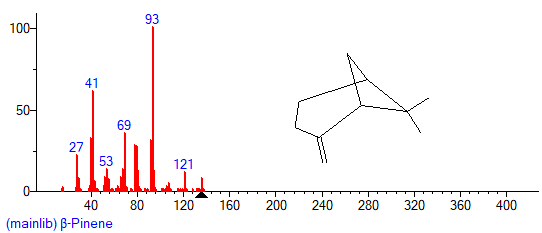 | | 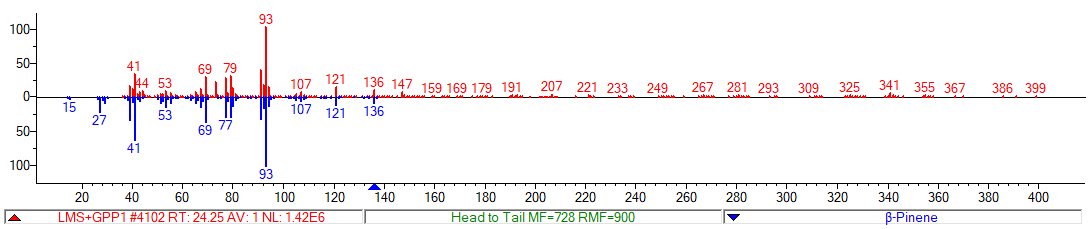 |  |
|  |  | 25.91 | limonene | | 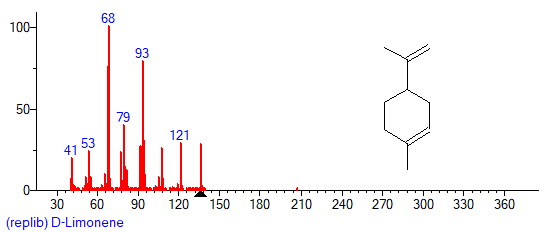 | | 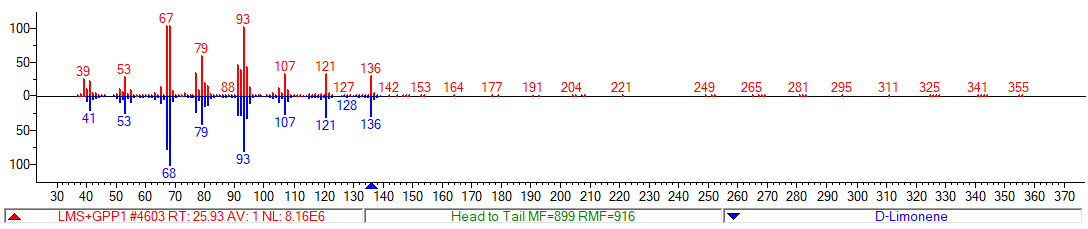 |  |
|  |  | 27.9 | terpinolene | | 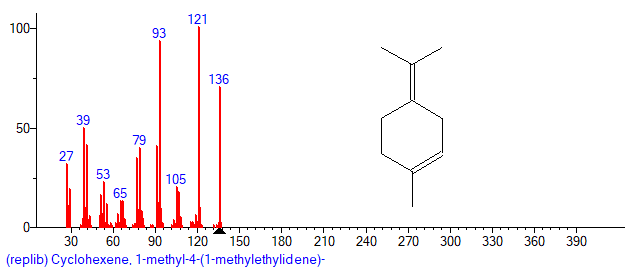 | | 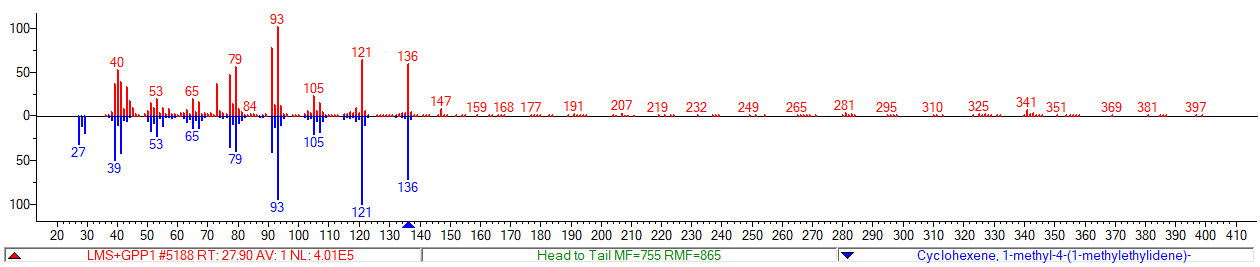 |  |
|  |  | 29.25 | fenchol* | | 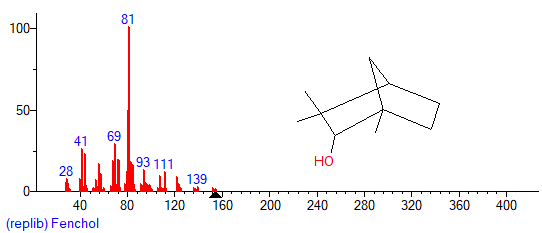 | | 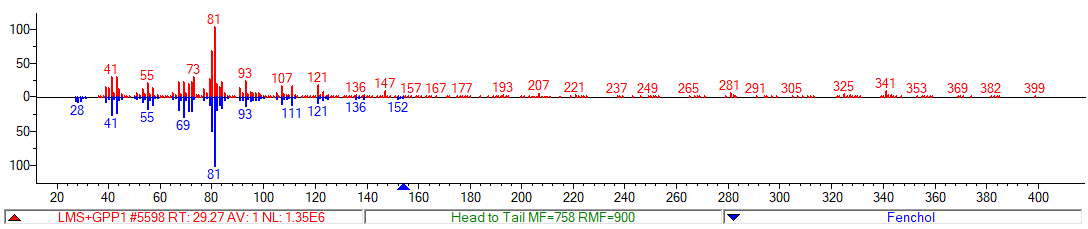 |  |
|  |  | 29.57 | β-terpineol* | | 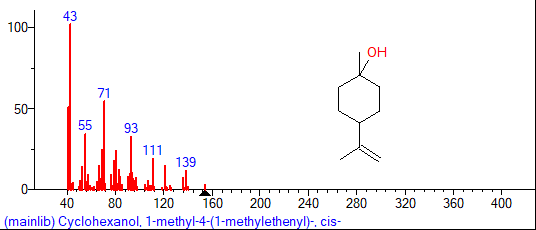 | | 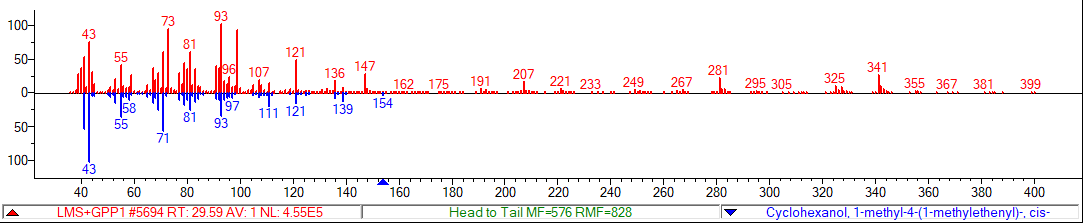 |  |
|  |  | 31.58 | α-terpineol | | 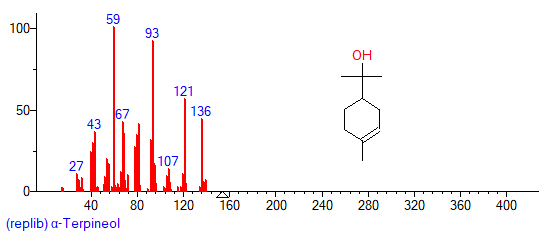 | | 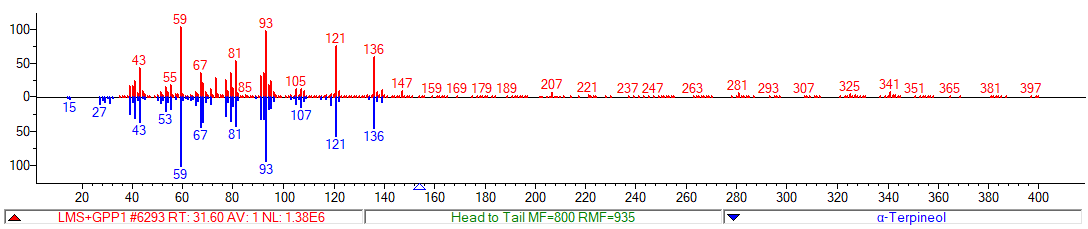 |  |
|  |  | 32.8 | geraniol | | 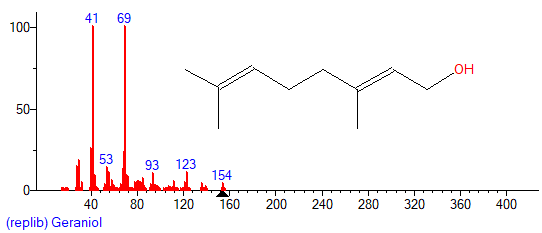 | | 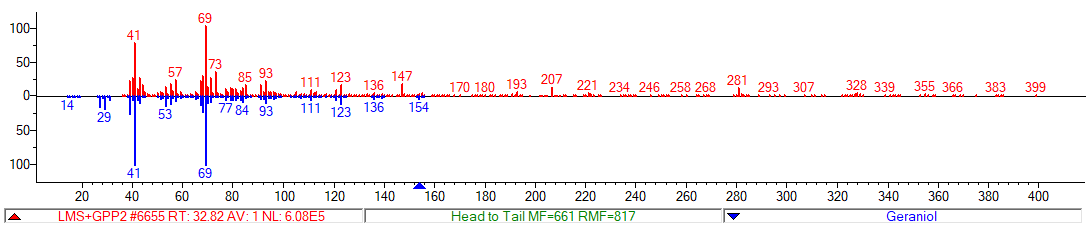 |  |
| CsTPS13PK | | 25.66 | (E)-β-ocimene | | 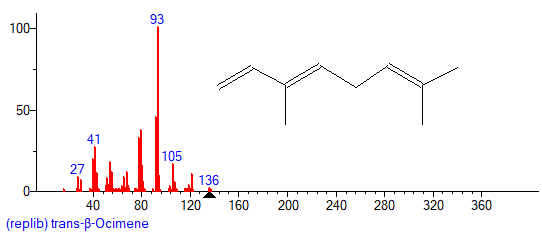 | | 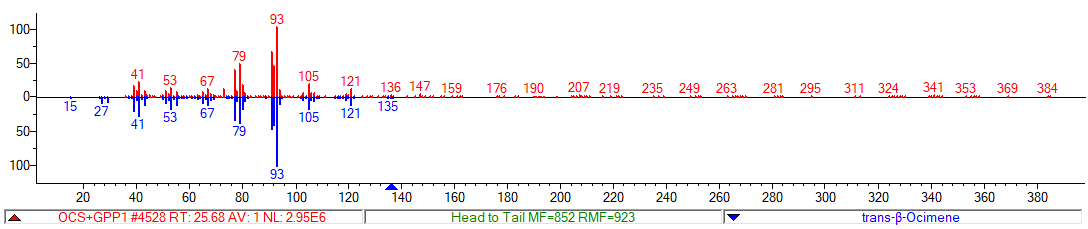 |  |
|  |  | 26.1 | (z)-β-ocimene | | 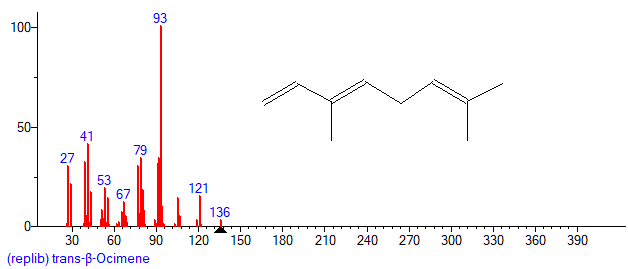 | | 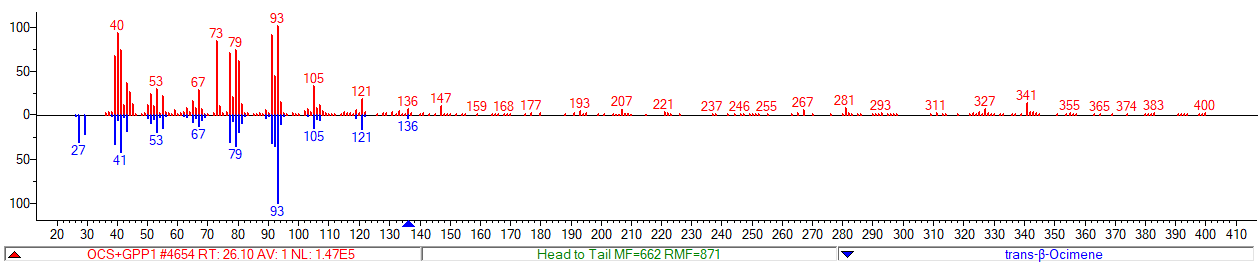 |  |
|  |  | 28.94 | allo-ocimene* | | 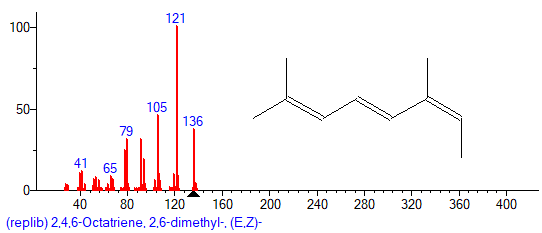 | | 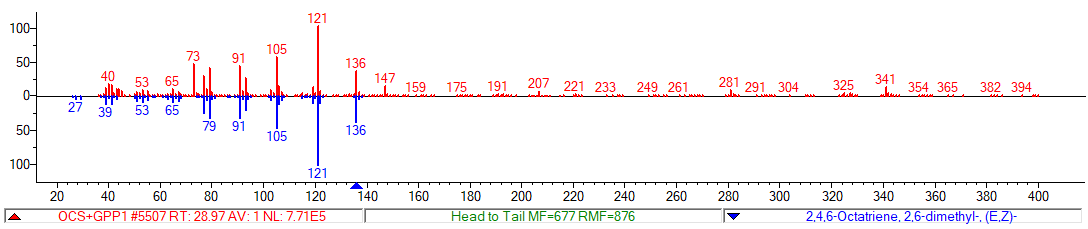 |  |
| CsTPS37FN | | 22.42 | α-pinene | | 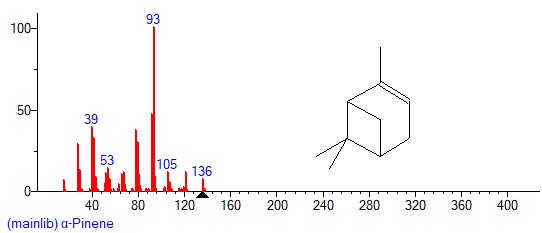 | | 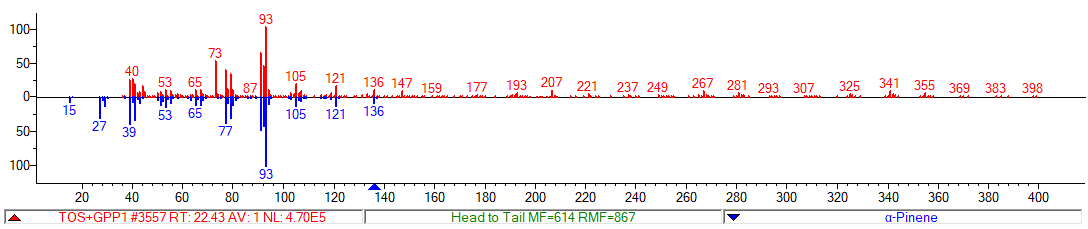 |  |
|  |  | 23.82 | β-phellandrene* | | 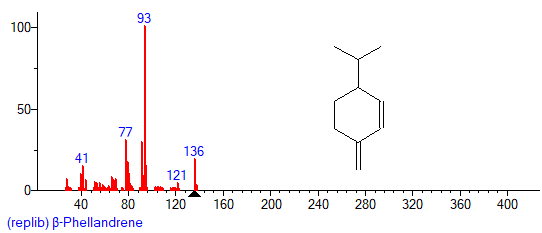 | | 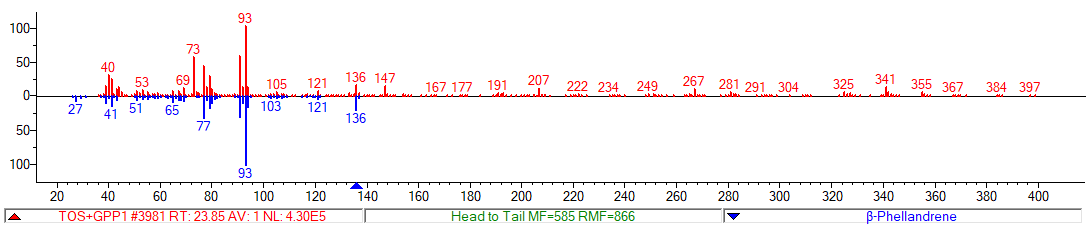 |  |
|  |  | 24.04 | β-myrcene | | 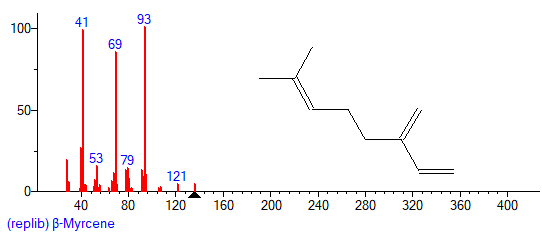 | | 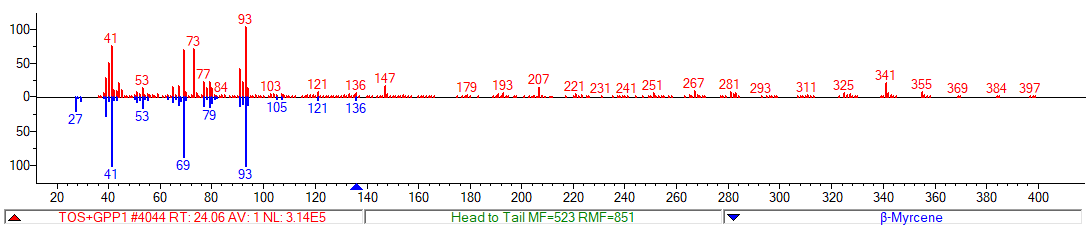 |  |
|  |  | 24.23 | β-pinene | | 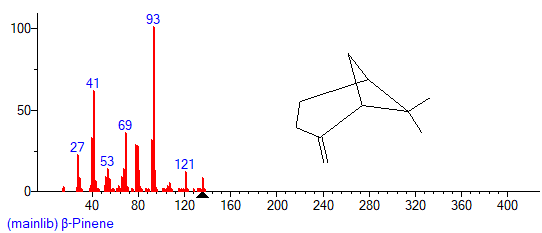 | | 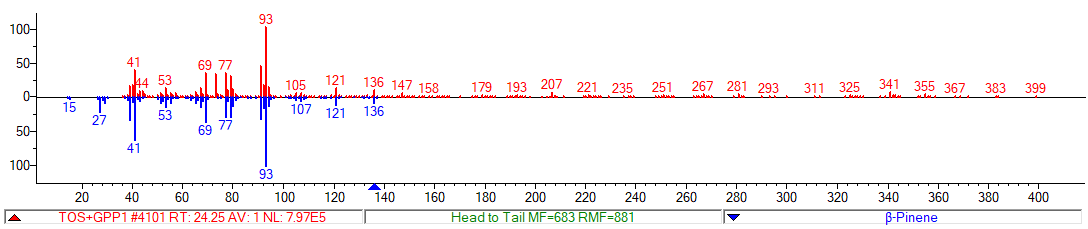 |  |
|  |  | 25.25 | ∆-carene | | 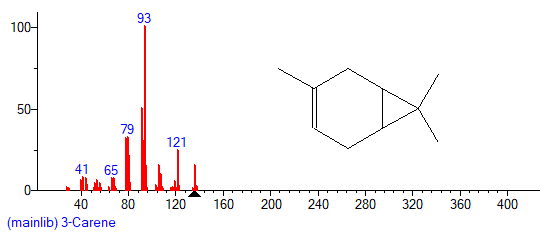 | | 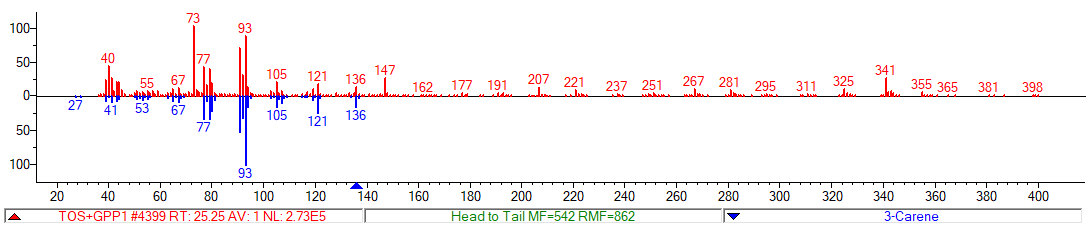 |  |
|  |  | 25.45 | α-terpinene | | 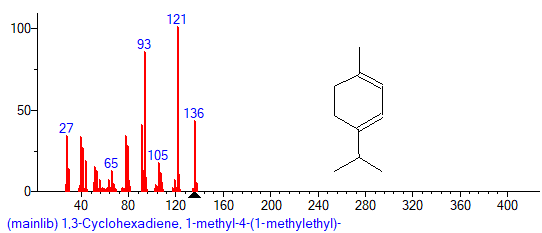 | | 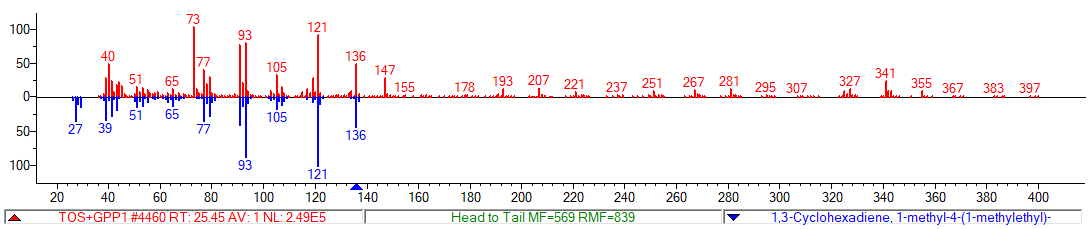 |  |
|  |  | 25.92 | limonene | | 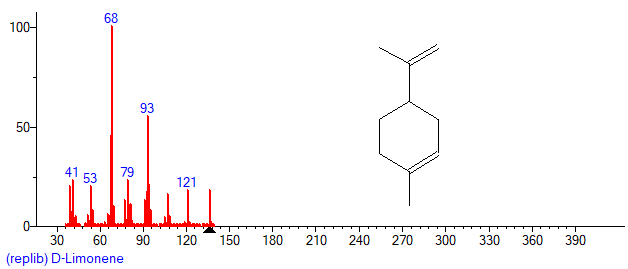 | | 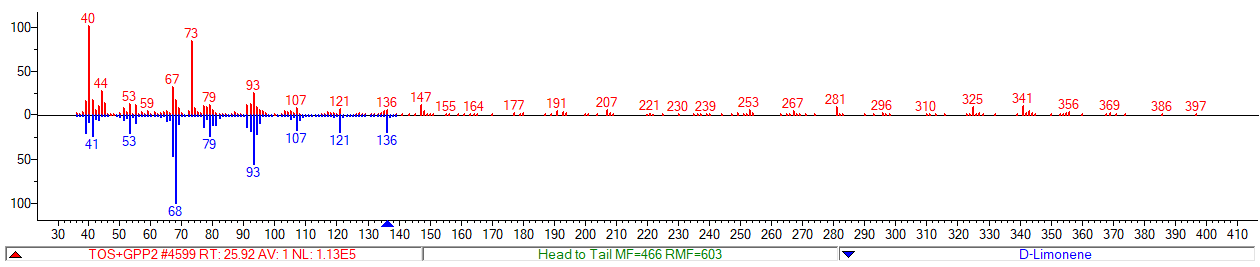 |  |
|  |  | 26.86 | y-terpinene | | 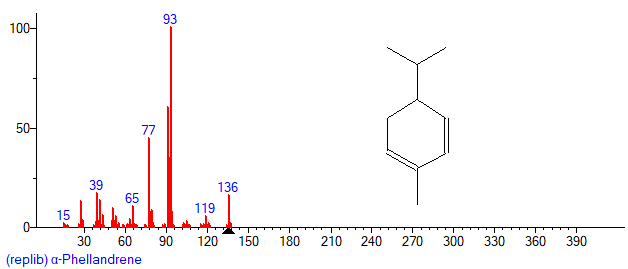 | | 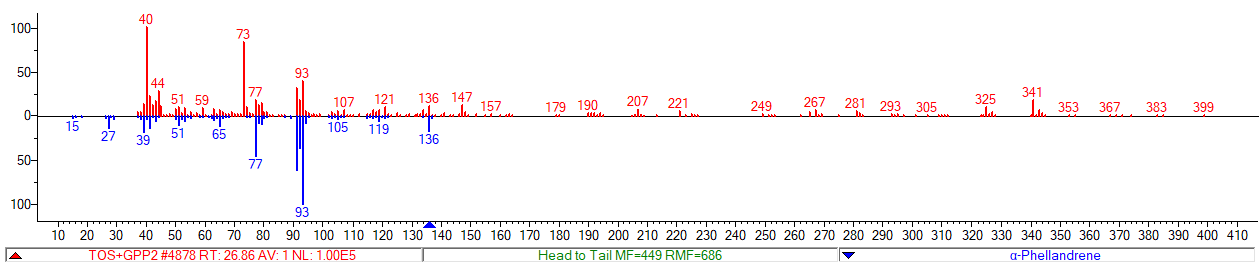 |  |
|  |  | 27.89 | terpinolene | | 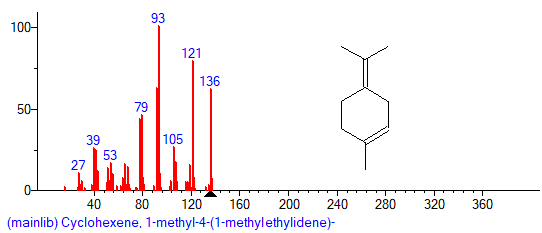 | | 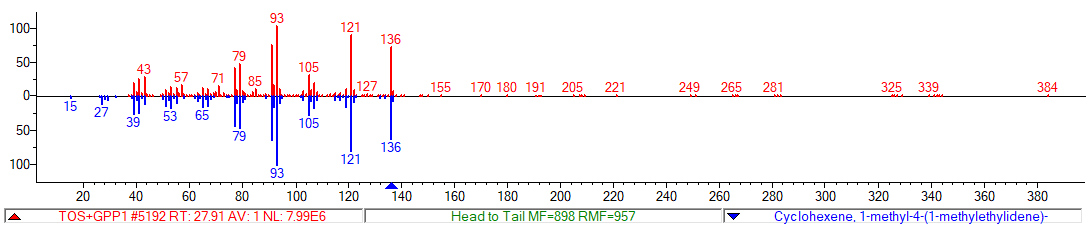 |  |
|  |  | 27.97 | linalool | | 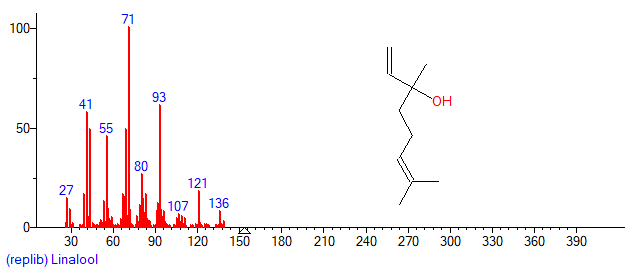 | | 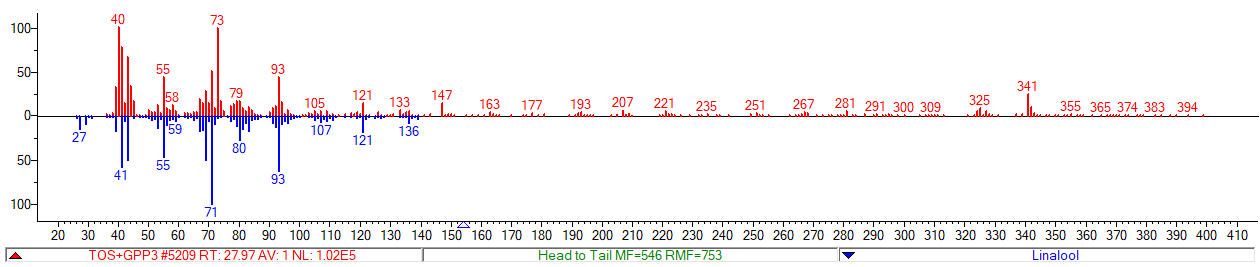 |  |
|  |  | 32.8 | geraniol | | 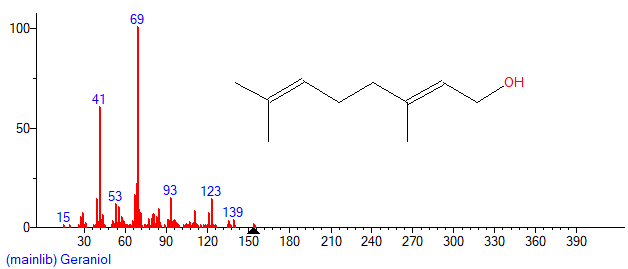 | | 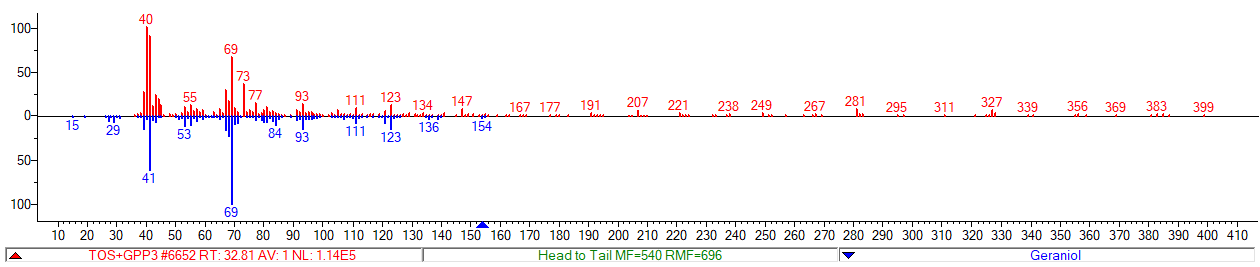 |  |
| CsTPS19BL | | 27.98 | linalool | | 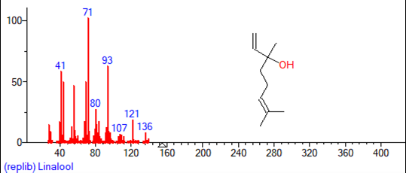 | | 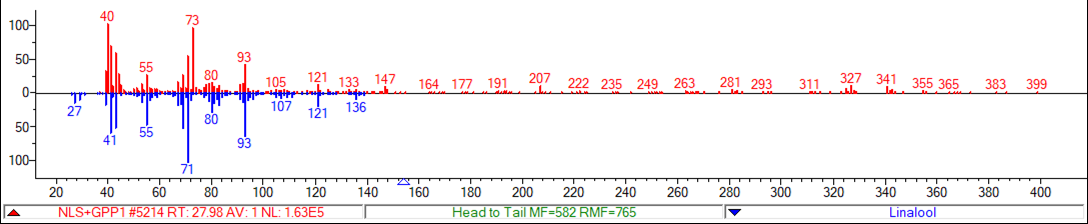 |  |
| CsTPS12PK | | 24.07 | β-myrcene | | NI | | NI |  |
|  |  | 25.44 | α-terpinene | | 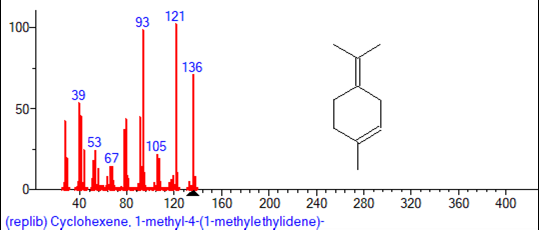 | | 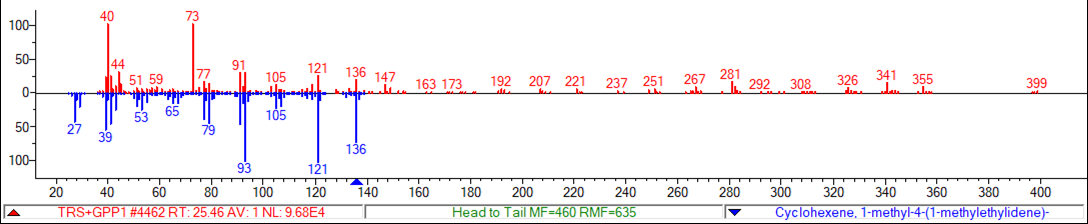 |  |
|  |  | 25.91 | limonene | | 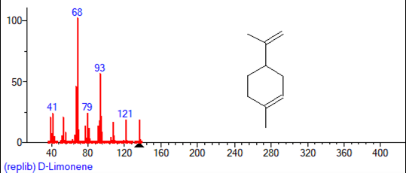 | | 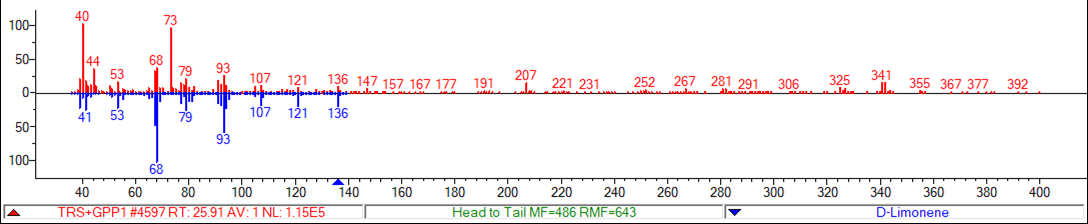 |  |
|  |  | 26.87 | γ-terpinene | | 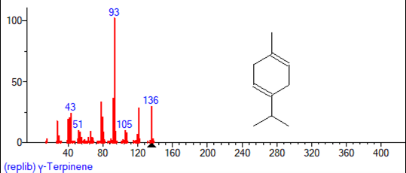 | | 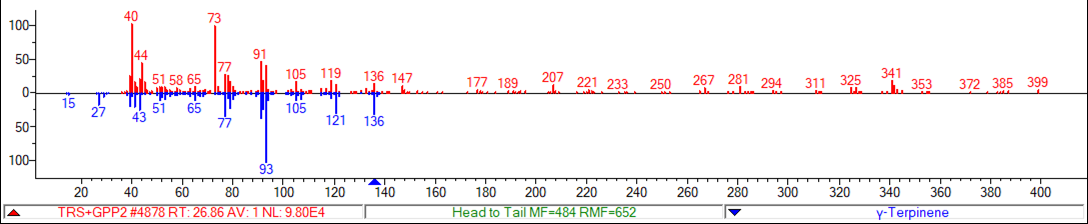 |  |

**Figure S4.** Terpene products identified in recombinant *Cannabis sativa* terpene synthase enzyme assays using Gas-chromatography mass-spectrometry (GC-MS). The figure shows retention times, identified terpene compounds, and their corresponding National Institute of Standards and Technology (NIST) library matches. For each compound, the most likely hit is provided, along with head-to-tail plots comparing the search spectrum with the reference spectrum from the NIST library. *No reference standard available, NI; Not identified
